# Who Provides Care in a Crisis? Family Caregivers’ Experiences of Home Care During the COVID-19 Pandemic

**DOI:** 10.1093/geroni/igab046.2058

**Published:** 2021-12-17

**Authors:** Jennifer Reckrey, Sasha Perez, Katherine Ornstein

**Affiliations:** 1 Icahn School of Medicine at Mount Sinai, Icahn School of Medicine at Mount Sinai, New York, United States; 2 Icahn School of Medicine at Mount Sinai Hospital, Icahn School of Medicine at Mount Sinai, New York, United States; 3 Icahn School of Medicine at Mount Sinai, New York, New York, United States

## Abstract

Many homebound individuals with dementia rely on both paid caregivers (e.g., home health aides, home attendants, other homecare workers) and family caregivers to live safely at home. We conducted semi-structured interviews with 15 family caregivers of individuals with severe dementia receiving home-based primary care in NYC to explore how caregiving changed during the COVID pandemic. Most individuals with long-standing paid caregivers experienced infrequent home care disruptions. In fact, paid caregivers were often the primary and sometimes only individuals to provide direct care; family caregivers themselves often stayed away and managed care from a distance. While most family caregivers described heightened attention to infection control, guidance about COVID prevention and safety rarely came from home-based primary care providers or home care agencies and instead was considered “common sense.” These findings confirm the essential role paid caregivers play in home-based dementia care teams.

